# Changes of cerebrospinal fluid pressure gradient in different body positions under experimental impairment of cerebrospinal fluid pathway: new insight into hydrocephalus development

**DOI:** 10.3389/fnmol.2024.1397808

**Published:** 2024-06-14

**Authors:** Ivana Jurjević, Darko Orešković, Milan Radoš, Klara Brgić, Marijan Klarica

**Affiliations:** ^1^Department of Pharmacology and Croatian Institute for Brain Research, School of Medicine, University of Zagreb, Zagreb, Croatia; ^2^Department of Neurology, University Hospital Centre Zagreb, Zagreb, Croatia; ^3^Department of Molecular Biology, Ruđer Bošković Institute, Zagreb, Croatia; ^4^Department of Neurosurgery, Univesity Hospital Centre Zagreb, Zagreb, Croatia

**Keywords:** CSF pressure gradient, body position, aqueductal obstruction, cervical stenosis, hydrocephalus

## Abstract

It is generally accepted that hydrocephalus is a consequence of the disbalance between cerebrospinal fluid (CSF) secretion and absorption which should in turn lead to CSF pressure gradient development and ventricular enlargement. To test CSF pressure gradient role in hydrocephalus development, we experimentally caused CSF system impairment at two sites in cats. In the first group of animals, we caused Sylvian aqueduct obstruction and recorded CSF pressure changes pre and post obstruction at three measuring sites (lateral ventricle -LV, cortical-CSS and lumbar subarachnoid space -LSS) during 15 min periods and in different body positions over 360 degrees. In the second group of experiments, we caused cervical stenosis by epidural plastic semiring implantation and monitored CSF pressure changes pre and post stenosis implantation at two measuring sites (lateral ventricle and lumbar subarachnoid space) during 15 min periods in different body positions over 360 degrees. Both groups of experimental animals had similar CSF pressures before stenosis or obstruction at all measuring points in the horizontal position. During head-up verticalization, CSF pressures inside the cranium gradually became more subatmospheric with no significant difference between LV and CSS, as they are measured at the same hydrostatic level, while CSF pressure inside LSS became more positive, causing the development of a large hydrostatic gradient between the cranial and the spinal space. With cervical stenosis, CSF pressure inside the cranium is positive during head-up verticalization, while in cats with aqueductal obstruction CSF pressure inside the CSS remains negative, as it was during control period. Concomitantly, CSF pressure inside LV becomes less negative, thus creating a small hydrostatic gradient between LV and CSS. Since CSF pressure and gradient changes occur only by shifting body position from the horizontal plane, our results indicate that cervical stenosis in a head-up vertical position reduces blood perfusion of the whole brain, while aqueductal obstruction impairs only the perfusion of the local periventricular brain tissue. It seems that, for evolutionary important bipedal activity, free craniospinal communication and good spinal space compliance represent crucial biophysical parameters for adequate cerebral blood perfusion and prevention of pathophysiological changes leading to the development of hydrocephalus.

## 1 Introduction

In accordance to the classical hypothesis of cerebrospinal fluid (CSF) hydrodinamics (Davson et al., 1987; Pollay, 2010; Sakka et al., 2011; Brinker et al., 2014) hydrocephalus may develop as a result of an obstruction of the circulating pathways, a reduction in the ability to absorb the CSF, or by an overproduction of CSF. Hence, it is believed that the general mechanism of hydrocephalus development is an imbalance between the CSF formation and absorption where more CSF is formed than absorbed, which results in an abnormal increase of CSF volume and pressure inside the cranial CSF space. Obstruction of the CSF pathways somewhere between the hypothetical site of CSF formation and the site of its absorption is deemed as the main cause of the mentioned imbalance, diminishing or preventing CSF outflow from the cranial space (Milhorat, 1972; Hochwald, 1984; Pollay, 1984; Davson et al., 1987; McComb, 1989; Milhorat, 1989). In the case of CSF circulation obstruction, hydrostatic CSF pressure gradient develops between the ventricles and the subarachnoid space due to supposed accumulation of the newly formed CSF, which is instrumental for the rapid hydrocephalus development (so-called acute hydrocephalus) (Dandy, 1919; Milhorat, 1972; Hochwald, 1984; Pollay, 1984; Davson et al., 1987; McComb, 1989; Milhorat, 1989).

Thus, according to the classical concept, the mechanism of hydrocephalus development primarily involves active CSF production or overproduction by choroid plexuses, impaired circulation, decreased absorption and increased hydrostatic CSF pressure (Dandy, 1919; Di Chiro, 1964; Milhorat, 1972; Welch, 1975; Hochwald, 1984; Pollay, 1984; Davson et al., 1987; McComb, 1989; Milhorat, 1989; Pollay, 2010; Sakka et al., 2011; Brinker et al., 2014). However, many forms of hydrocephalus cannot be explained by this concept, mostly emphasising the specific forms of communicating hydrocephalus such as unilateral communicating hydrocephalus, transitory hydrocephalus and arrested or slow-progressing forms with normal pressure such as iNPH (Foltz and Shurtleff, 1966; Holtzer and de Lange, 1973; Di Rocco et al., 1978; Davis, 1981; Griffith and Jamjoom, 1990; Klarica et al., 2009, 2013, 2014; Krishnamurthy et al., 2009; Radoš et al., 2014a,b; Xi et al., 2014; Orešković et al., 2017; Jovanović et al., 2021). The concept also does not provide an adequate explanation of some forms of hydrocephalus which develop concomitantly with tumors or other pathological conditions situated inside the spinal space (Arseni and Maretsis, 1967; Raynor, 1969; Borgesen et al., 1977; Rifkinson-Mann et al., 1990; Cinalli et al., 1995; Morandi et al., 2006; Mirone et al., 2011; Wilson et al., 2013; Mukherjee et al., 2019; Mathkour et al., 2020), since these conditions do not physically interrupt free CSF movement from the hypothetical site of its secretion to the hypothetically dominant site of its absorption (dural sinuses arachnoid granulations) inside the cranium.

Additionally, it is not possible to explain the existence and even aggravation of hydrocephalus post plexectomy (Orešković and Klarica, 2011; Orešković, 2015). Many results of experimental studies on animals, as well as the results from clinical studies imply that there is also a substantial CSF absorption inside the brain ventricles (in both free communicating ventricles and those under obstruction or stenosis) and that significant CSF absorption from the spinal subarachnoid space also takes place (Orešković and Klarica, 2010; Bulat and Klarica, 2011; Orešković and Klarica, 2011; Klarica and Orešković, 2014). Some literature data imply that choroid plexuses and brain ventricles are not the main and exclusive sources of CSF (Bering, 1952; Orešković et al., 1991; Oreškovic et al., 2001; Orešković et al., 2002; Maraković et al., 2010; Klarica et al., 2013; Igarashi et al., 2014; Mehemed et al., 2014; Nakada, 2014; Orešković, 2015), i.e., that CSF can be formed and absorbed anywhere across the CNS capillary network under the gradient of hydrostatic and osmotic forces (Orešković et al., 1991; Bulat et al., 2008; Klarica and Orešković, 2014; Orešković and Klarica, 2014). It was also demonstrated that, in the case of completely patent CSF pathways, an experimental increase of osmolarity can result in hydrocephalus development (Krishnamurthy et al., 2012; Klarica et al., 2013). Ventriculomegaly formation was also observed during experimental subchronic cervical stenosis (Klarica et al., 2016). All of the aforementioned implies that it is necessary to reconsider former concepts of hydrocephalus development, especially those connected to CSF circulation obstruction within the entire craniospinal system and development of CSF pressure gradient. For this purpose, we have experimentally induced an obstruction inside the cranium and spinal stenosis of the CSF system in cats in order to investigate biophysical conditions of either hydrostatic CSF pressure increase or gradient formation by monitoring the changes of CSF pressure at various measuring sites and in different body positions.

## 2 Materials and methods

### 2.1 Experimental animals

The study was performed on a total of 9 adult cats (both sex; 2.2–3.4 kg body weight). The animals were obtained from private owners according to the old Croatian Animal Welfare Act which allowed us to obtain experimental animals from domestic breeding. However, today in Croatia we have a new Animal Welfare Act by which it is possible to obtain experimental animals only from official suppliers (and we are currently doing so). The owners were verbally informed about the experimental protocol which was previously approved by official Ethical committee (written consent form was not needed in that time). The animals were kept in cages with natural light–dark cycles and had access to water and food (SP215 Feline, Hill’s Pet Nutrition Inc., Topeka, KS, United States). They were in quarantine for 30 days prior to the experiments, and the procedures were performed in accordance with the Croatian Animal Welfare Act. The protocol was approved by the Ethics Committee of the University of Zagreb Medical School (Approval No. 04–76/2009–761). All efforts were made to minimize suffering, and all surgery according to protocol was performed under anesthesia. The cats were anaesthetized with a-chloralose (Fluka; 100 mg/ kg i.p.) and fixed in a stereotaxic head holder (David Kopf, Tununga, CA, United States) in the sphinx position. The femoral artery was cannulated, the blood pressure was recorded via a T-connector, and samples of blood were taken for analysis of the blood gases. No significant changes, either in blood pressure or blood gases, were observed during these experiments on cats, which continued breathing spontaneously under the chloralose anesthesia. At the end of experiment the animals were sacrified with an excessive dose of anesthesia (thiopental).

### 2.2 Surgical procedure

**Figure 1:**
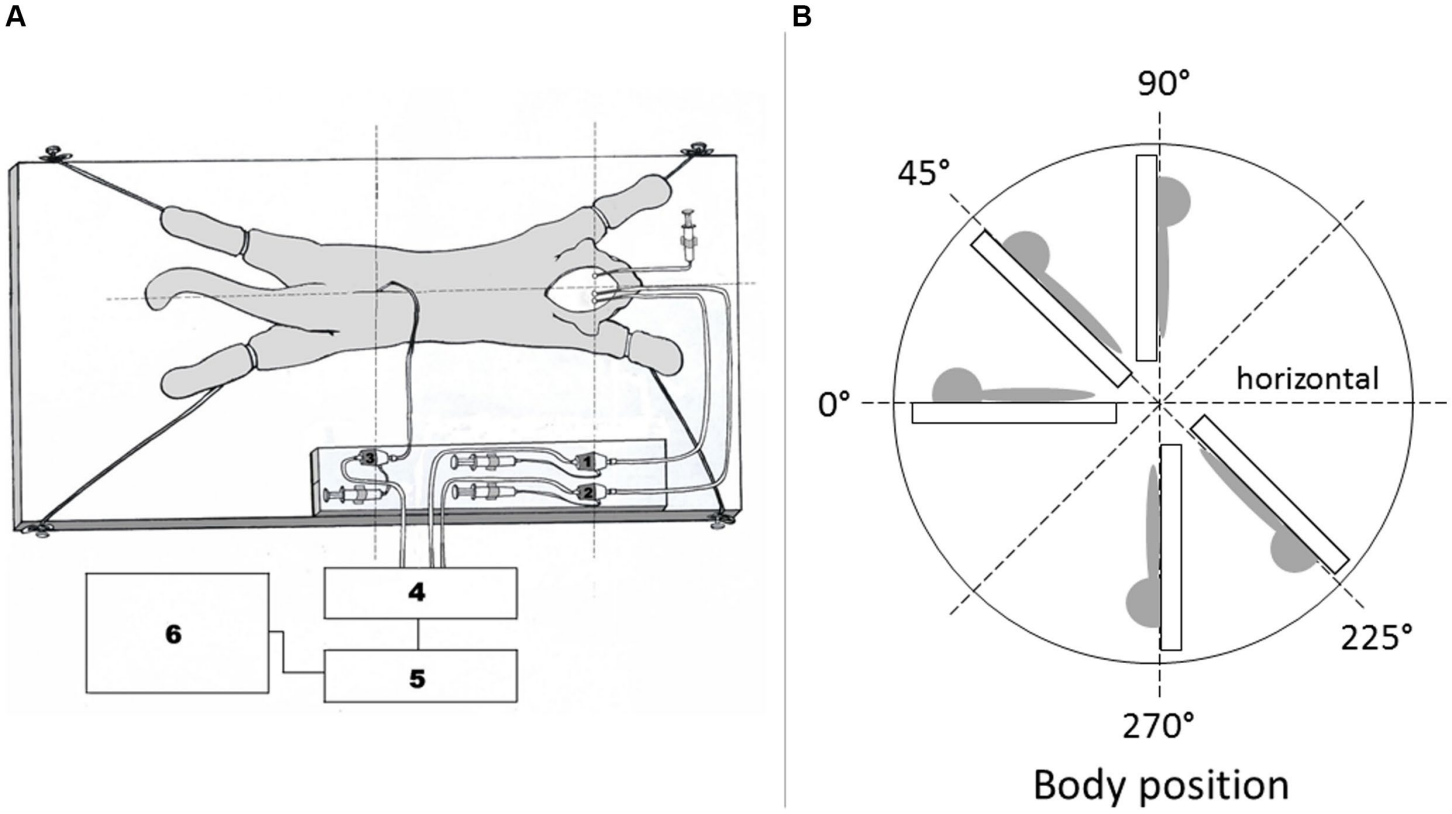
**(A)** Scheme of a cat experimental model. The animal is fixed onto the flat board, together with pressure tranducers and measuring cannulas. 1 – pressure transducer connected to the measuring cannula inside the right lateral ventricle, 2 – pressure transducer connected to the measuring cannula inside the cortical subarachnoid space, 3 – pressure transducer connected to the measuring cannula inside the lumbar subarachnoid space. 4 – Quad Bridge, 5 – PowerLab/800, AD Instruments, Castle Hill, NSW, Australia, 6 – computer. Syringe is connected to cannula in left lateral ventricle. **(B)** Schematic display of the body position changes in which CSF pressure measurements were performed. CSF pressure was measured inside the lumbar and cortical subarachnoid space, as well as in the lateral ventricle of experimental animals in horizontal position 0°, head- up position 45°, head-up position 90°, head-down position 225°, and head-down position 270°.

Surgical procedure and position of animal during recording CSF pressure are previously described in detail (Klarica et al., 2014, 2016). In short, the anesthetized cats were set into the stereotaxic device in a sphynx position. A stainless steel cannula (0.9 mm ID) was introduced into the left lateral ventricle (LV; cannula with siringe in Figure 1A) at 2 mm lateral and 15 mm anterior to the stereotaxic zero point, and 10–12 mm below the dural surface. A second cannula was placed in the right lateral ventricle (LV) (at same position as the cannula in the left LV). A third cannula was then placed into the cortical subarachnoid space (CSS) at the same hydrostatic level as the cannula placed in the LV when the body is in vertical position. In horizontal body position cannula in CSS is about 0.5 cm above the cannula in LV. The cannulas in the right LV and the CSS were used for the measurement of intracranial CSF pressures. In order to measure the spinal CSF pressure in the lumbar region, a laminectomy (5.0 × 10.0 mm) of the L3 vertebra was performed. After incision of the spinal dura and arachnoidea, a fourth plastic cannula (0.9 mm ID) was introduced into the lumbar subarachnoid space (LSS). Leakage of CSF was prevented by applying cyanoacrylate glue to the dura around the cannula. Bone openings in the cranium and vertebra were hermetically closed by the application of a dental acrylate. After setting the measuring cannulas, the cat was removed from the stereotaxic device and then fixed in a prone position on a board (Figure 1A). CSF pressures were recorded using pressure transducers (Gould P23 ID, Gould Instruments, Cleveland, OH, United States) which were connected to a system that transformed analogous to digital data (Quand Bridge and PowerLab/800, ADInstruments, Castle Hill, NSW, Australia), and then entered into a computer (IBM, White Plains, NY, United States). Pressure transducers were calibrated by use of a water column; the interaural line was taken as zero pressure. Instruments for pressure measurement were fixed on the board in such a way that the membrane of each transducer was at the same hydrostatic level as the corresponding measuring cannula, so there was no need to additionally adjust the transducers during the body position changes. The cats were also fixed onto the measuring board by their extremities and their head to avoid any movement during body position changes. This enabled us to maintain the same distance of the measuring cannulas and the pressure tranducers according to their real hydrostatic level in any body position, thus giving us an opportunity to examine how the CSF pressure changes in relation to the distance of the measuring cannulas from cisterna magna.

### 2.3 Basic measurements during control period (normal CSF pathway)

According to RRR principals each experimental animal (*n* = 9) also served for control measurements of CSF pressure. During that control period we measured CSF pressures inside the LV, CSS and LSS in different body positions without any CSF pathway impairment (horizontal 0°, head-up 45°, vertical head-up 90°, head-down 225°, and vertical head-down 270°) (Figure 1B). CSF pressure changes were recorded at 15 min intervals.

### 2.4 Blockade of the Sylvian aqueduct

**Figure 2:**
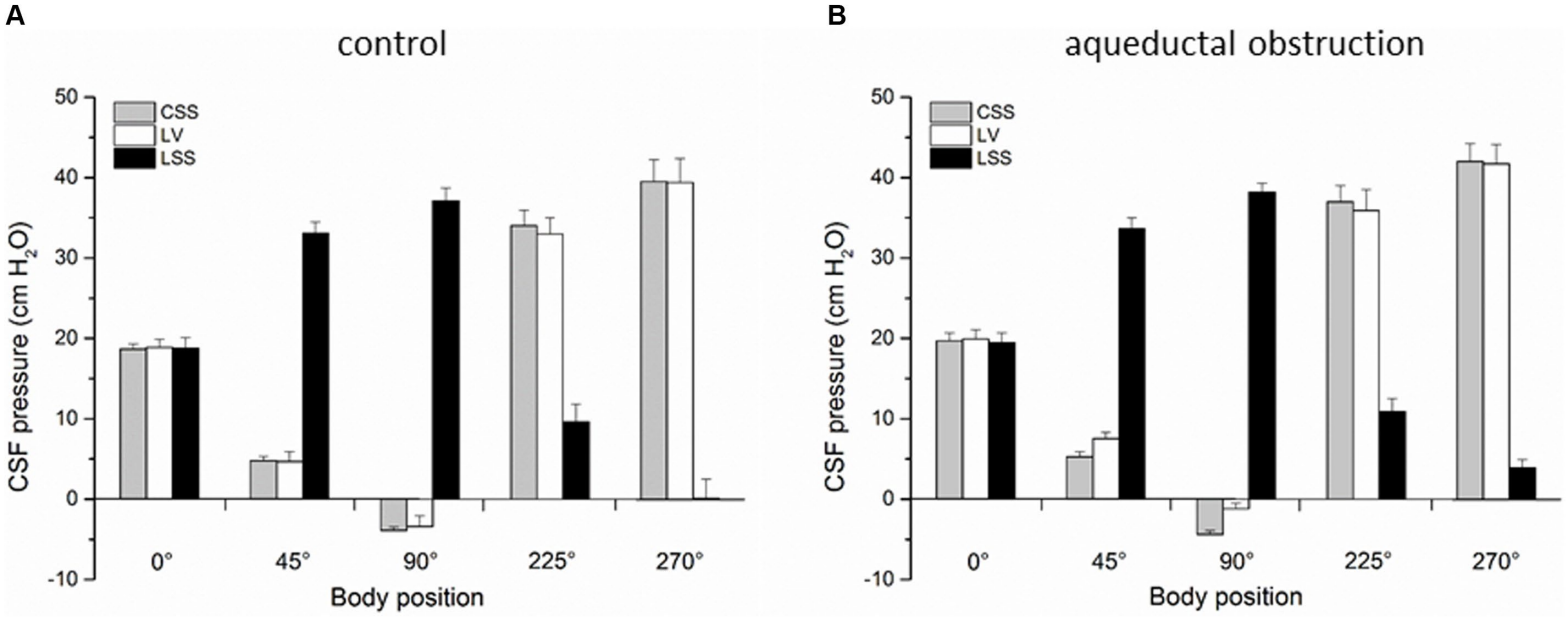
**(A)** Changes of CSF pressure (cm H_2_O) inside the lateral ventricle (LV, 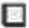), cortical (CSS, 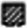) and lumbar (LSS, 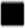) subarachnoid space in horizontal position (0°), head-up position (45°), head-up position (90°), head-down position (225°), and head-down position (270°) in cats (*n* = 4) with open CSF pathway (control). **(B)** Changes of CSF pressure inside the lateral ventricle (LV, 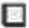), cortical (CSS, 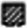) and lumbar (LSS, 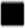) subarachnoid space in horizontal position (0°), head-up position (45°), head-up position (90°), head-down position (225°), and head-down position (270°) in cats (*n* = 4) with aqueductal obstruction. The columnes represent the mean values, and the vertical marks are standard errors of the mean.

In the first group of experiments on anesthetized cats (*n* = 4) after control period of CSF pressure measurement we performed occipital craniectomy followed by tunneling of the cerebellar vermis right up to the entrance into the Sylvian aqueduct. A plastic cannula with the diametar corresponding to the aqueductal width was carefully manually inserted through the tunnel in order to avoid the damage of the surrounding tissue. A drop of cyanoacrylate glue (Super Attack glue) was used to fix the cannula to the brain tissue to completely block the aqueduct. After reconstructing the bone and hermetically closing the surgical wound we proceeded to introduce the measuring cannulas into the LV and CSS as was previously described, followed by measurments of CSF pressures in different body positions, as mentioned above (Figure 2B).

### 2.5 Cervical stenosis

**Figure 3:**
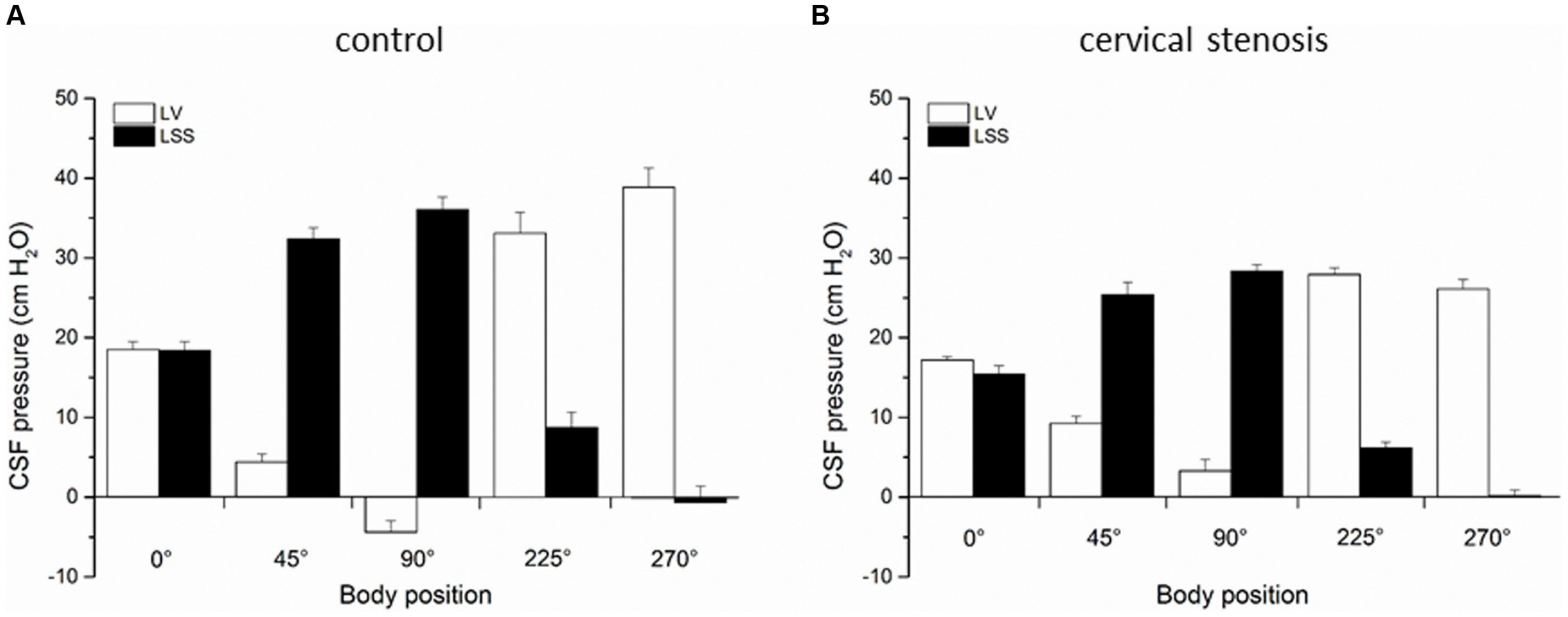
**(A)** Changes of CSF pressure (cm H_2_O) inside the lateral ventricle (LV, 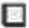), and lumbar (LSS, 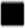) subarachnoid space in horizontal position (0°), head-up position (45°), head-up position (90°), head-down position (225°), and head-down position (270°) in cats (*n* = 5) with normal CSF pathway communication (control). **(B)** Changes of CSF pressure inside the lateral ventricle (LV, 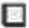), and lumbar (LSS, 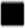) subarachnoid space in horizontal position (0°), head-up position (45°), head-up position (90°), head-down position (225°), and head-down position (270°) in cats (*n* = 5) with cervical stenosis. The columnes represent the mean values, and the vertical marks are standard errors of the mean.

In the second group of experiments (*n* = 5) on anesthetized cats after control period of CSF pressure recording we performed additional laminectomy of the cervical C2 vertebrae (5.0 ×10.0 mm) exposing the dura. Immediately after opening, a plastic semiring (width 2.0 mm; length 10.0 mm; thickness 1.0 mm) covering the dorsal and lateral parts of the dura and gently pressing on the cord was positioned in order to disable the communication between the cranial and the spinal subarachnoid space [details previously described in Klarica et al. (2014, 2016)]. We swiftly covered the opening with dental acrylate, that way hermetically isolating the system from the atmospheric pressure influence. The CSF pressures were then measured in the ventricles and in the lumbar subarachnoid space in different body positions, as described before (Figure 3B).

### 2.6 Statistical analysis

Data are shown as a mean value ± standard error of the mean (SEM). A statistical analysis of all of the results was performed using the Paired Student’s *t*-test and ANOVA for repeated measures. All statistical analysis was performed using the Statistica 7.1 (StatSoft Inc., Tulsa, OK, United States). *p* < 0.05 was considered as statistically significant.

## 3 Results

In the first group of experiments control results were obtained on 4 cats in which CSF pressure changes were observed inside the lateral ventricle (LV), cortical (CSS) and lumbar subarachnoid space (LSS) before surgical impairment of the Sylvian aqueduct. CSF pressures were measured during body position changes, as described above. Figure 2A shows that in horizontal position there is no statistical difference between the pressures measured inside the LV (18.9 ± 1.0 cm H_2_O), CSS (18.7 ± 0.6 cm H_2_O) and LSS (18.8 ± 1.3 cm H_2_O). During head-up position 45°, CSF pressure inside the lumbar space gradually increases to 33.1 ± 1.4 cm H_2_O and in vertical head-up position 90° it reaches 37.1 ± 1.6 cm H_2_O, while the cranial CSF pressure gradually decreases and amounts to 4.7 ± 1.2 cm H_2_O inside LV and 4.8 ± 0.5 cm H_2_O inside CSS during head-up position 45°, decreasing further to negative values in vertical head-up position 90° (−3.4 ± 1.3 cm H_2_O inside LV and −3.9 ± 0.4 cm H_2_O inside CSS). CSF pressures inside the cranium do not significantly differ and they are measured at the same hydrostatic level. In head-down position 225° CSF pressure inside LSS decreases to 9.6 ± 2.2 cm H_2_O and in head-down position 270° it was 0.1 ± 2.4 cm H_2_O, while the cranial CSF pressure gradually increases in head-down position 225° to 33.0 ± 2.2 cm H_2_O inside the LV and 34.0 ± 1.9 cm H_2_O inside CSS, further increasing in head-down position 270° to 39.4 ± 3.0 cm H_2_O inside the LV and 39.5 ± 2.7 cm H_2_O inside CSS. Again, there is no statistical difference between cranial pressures in these positions (Figure 2A).

In the second group of experiments done on the same cats (*n* = 4) described above, corresponding measurements of CSF pressures were performed inside the LV, CSS and LSS after blockade of the Sylvian aqueduct by insertion of a plastic tube matching in diametar to the width of the aqueduct. Figure 2B shows once more that the LV, CSS and LSS pressures are approximately the same (LV = 19.9 ± 1.2 cm H_2_O; CSS = 19.7 ± 1.0 cm H_2_O; LSS = 19.5 ± 1.2 cm H_2_O) and without statistically significant difference (*p* = 0.91). However, in an upright position LV and CSS pressures are not equally negative any more, i.e., CSF pressure inside the LV is now more positive than the one inside the CSS, measuring 7.5 ± 0.8 cm H_2_O in the head-up position 45° and −1.2 ± 0.7 cm H_2_O in the head-up position 90°, while the CSF pressure inside the CSS remains equally low as in the case of open aqueduct, amounting to 5.3 ± 0.6 cm H_2_O in head-up position 45°, while it is −4.4 ± 0.5 cm H_2_O in head-up position 90°. Thus, during animal verticalization to head-up position 90° a statistically significant difference between the CSF pressure inside LV and CSS can be observed (*p* = 0.01). At the same time, CSF pressure inside the LSS increases again to positive values and measures 33.6 ± 1.4 cm H_2_O in head-up position 45° and 38.2 ± 1.1 cm H_2_O in head-up position 90°. In the head-down position 225° CSF pressure lumbally decreases to 10.9 ± 1.6 cm H_2_O, and to 3.9 ± 1.0 cm H_2_O in head-down position 270°, while the CSF pressures inside the LV and CSS rise again to equally positive values, amounting to 35.9 ± 2.6 cm H_2_O in head-down position 225° and to 41.7 ± 2.4 cm H_2_O in head-down position 270° in LV, while it was 37.0 ± 2.0 cm H_2_O in head-down position 225° and 42.0 ± 2.2 cm H_2_O in head-down position 270° in CSS (Figure 2B).

The third group of experiments was done as control measurements on cats (*n* = 5) before insertion of cervical stenosis. CSF pressure changes were measured inside the lateral ventricle LV, CSS and LSS in different body positions, as described before. Figure 3A shows again that the pressures inside the LV and LSS do not differ in horizontal position (LV = 18.5 ± 1.0 cm H_2_O, LSS = 18.4 ± 1.1 cm H_2_O) and are not statistically significantly different, while in the head-up position lumbar pressure gradually increases, amounting to 32.4 ± 1.3 cm H_2_O in head-up position 45° and 36.0 ± 1.6 cm H_2_O in head-up position 90°. The pressure inside the cranial LV simultaneously drops to negative values, and measures 4.4 ± 1.0 cm H_2_O in head-up position 45° and −4.4 ± 1.4 cm H_2_O in head-up position 90°. During the cat head-down position, LSS pressure gradually becomes negative and in the head-down position 225° it is 8.7 ± 1.9 cm H_2_O, while in the head-down position 270° it is −0.7 ± 2.0 cm H_2_O. At the same time, pressure inside LV increases to 33.1 ± 1.6 cm H_2_O in the head-down position 225° and to 38.9 ± 2.9 cm H_2_O in the head-down position 270° (Figure 3A).

In the last experimental series, an insertion of cervical stenosis was performed in cats (*n* = 5), as described in Materials and methods. We then performed measurements of CSF pressures inside the LV and LSS in aforementioned various body positions. Figure 3B shows that in horizontal position pressures inside the LV and LSS are still equal (LV = 17.2 ± 0.4 cm H_2_O; LSS = 15.5 ± 1.0 cm H_2_O) and without significant statistical difference (*p* = 0.239). However, LV pressure now remains positive in both head-up position 45° (9.2 ± 0.9 cm H_2_O) and in head-up position 90° (3.3 ± 1.4 cm H_2_O), while LSS pressure increases again to even more positive values in both head-up position 45° (25.4 ± 1.6 cm H_2_O) and in head-up position 90° (28.3 ± 0.8 cm H_2_O). Thus, compared with the control measurements done on the same animals before insertion of cervical stenosis in the same body positions, LV pressure differs significantly post cervical stenosis (in the head-up position 45° *p* = 0.0075, in the head-up position 90° *p* = 0.0054). During the cat head-down position, CSF pressure inside the LV increases, and amounts to 27.9 ± 1.8 cm H_2_O at 225° and to 26.1 ± 1.2 cm H_2_O at 270°, while LSS pressure decreases, measuring 6.1 ± 0.8 cm at 225° and 0.1 ± 0.8 cm H_2_O at 270° (Figure 3B).

## 4 Discussion

### 4.1 CSF pressure changes inside the cranium during head-up verticalization

Results of CSF pressure measurement inside the LV, CSS and LS of control animals (no obstruction or stenosis) during the changes of body position were in accordance with previously published results (Kuzman et al., 2012; Klarica et al., 2014, 2022). Namely, during gradual head-up verticalization CSF pressure inside the cranium progresivelly dropped to subatmospheric (negative) values in the vertical head-up position 90° (Figures 2A, 3A). CSF pressures inside the LV and CSS were around −4 cm H_2_O, which corresponded to the hydrostatic distance from the measuring cannulas inside the LV and CSS to foramen magnum (Figure 2A). In accordance with the law of fluid mechanics, hydrostatic pressure can be calculated anywhere within the system if the distance from the reference point is known (P = ρ x g x h, where P is the pressure, ρ is the fluid density, g is the gravitational force, and h is the height of fluid column), and by that it is possible to explain the changes in pressure at different body positions. All of this suggests that CSF pressure changes during the changes of body position do not depend on CSF secretion, circulation and absorption, as was explained in detail in previous publications (Klarica et al., 2014, 2022).

In addition, Sylvian aqueduct obstruction during head-up verticalization caused CSF pressure inside the LV to decrease more slowly, so in vertical head-up position 90° pressure inside the LV was less negative and amounted to −1 cm H_2_O (Figure 2B). At the same time, CSF pressure inside the CSS gradually decreased as in control conditions (there was normal CSF communication between CSS and LSS), and in vertical head-up position 90° it was about −4 cm H_2_O, which led to development of a slight gradient between LV and CSS (Figure 2B). With aqueductal obstruction, CSF pressures inside the LV, CSS and LSS are equal in horizontal position 0° (Figure 2B) and pressure gradient between LV and CSS only starts forming after the change of body position. According to the classical concept of CSF secretion inside the ventricles, it would be expected that ventricular obstrucion resulted in an increased CSF pressure inside the ventricles, regardless of the position of the head toward the rest of the body. Thus, it is only during bipedal walking that aqueductal obstruction would be able to cause a lesser change of the CSF pressure inside the ventricles compared to the pressure change that occurs inside the subarachnoid space, which would potentially create a pressure gradient that inhibits blood perfusion of the brain tissue surrounding the ventrilces (compared to the more adequate perfusion of the cortical gray matter), further enabling the right biopyhsical conditions for the beginning of hydrcoephalus development.

Contrary to that, cervical stenosis during head-up verticalization caused even greater delay in CSF pressure decrease inside the LV compared to Sylvian aqueduct obstruction, and it finally remained on the positive level of +4 cm H_2_O which represents the hydrostatic distance between the measuring cannula inside the LV and foramen magnum (Figure 3B). In the horizontal position, cervical stenosis caused a slight difference between CSF pressures inside the LV and LSS, however, that difference significantly increased during head-up verticalization. Thus, in this case verticalization changed CSF pressure inside the LV by 8 cm H_2_O (from −4 cm H_2_O to +4 cm H_2_O) which also led to significantly decreased cerebral perfusion pressure (CPP). These results clearly indicate that during bipedal walking with normal CSF space communication CPP is much higher than it was previously believed (CSF pressure values are subatmospheric), and that different pathological processes which impair cranio-spinal communication (e.g., Arnold-Chiari malformation, tumours and other conditions that produce mass-effect inside the spinal space) significantly reduce CPP. It was previously believed that appearance of low or negative CSF pressure during head-up body verticalization is a transitory phenomenon caused by a slight and brief shift of CSF and blood from hydrostatically higher to hydrostatically lower compartments under the influence of gravity (Davson, 1967; Magnaes, 1976a,b; Davson et al., 1987). Shortly after (in a few minutes) it should go back to positive values due to hypothetical continuous CSF formation (Davson, 1967; Magnaes, 1976a,b; Davson et al., 1987). However, many clinical and animal research data imply that CSF pressure values are stabile as long as the body remains in a certain position, whether horizontal or head-up. Namely, in patients whose CSF pressure was measured for 60 min sitting up, it was constantly at the atmospheric level (zero CSF pressure) in the upper cervical region and at the same time positive inside the lumbar region, while the pressure values corresponded to the distance in cm from cisterna magna to the site of measurement in the lumbar part (Magnaes, 1976a,b). Similar results were obtained during intracranial pressure measurements in patients whose bodies were gradually verticalized (Chapman et al., 1990).

In our previous research, we kept experimental animals in a head-up position from 75 to 150 min and during that entire measuring period CSF pressure recorded inside the LV in those animals remained steadily negative (Klarica et al., 2014). It seems that appearance of negative CSF pressure is not a transitory phenomenon. Thus, although in this research we measured CSF pressure for a period of 15 min in one position before and after obstruction, we would expect for CSF pressure to remain equal and stabile even if we measured it for a longer period of time. Even in the case of aqueductal obstruction lasting 120 min (Klarica et al., 2009) or cervical stenosis over a period of 60 min (Klarica et al., 2016) in our previous work CSF pressure values inside the cranium measured in the spynx position were unchanged.

### 4.2 Changes of cranial and spinal CSF pressure during head-up verticalization

With normal CSF pathway communication (control animals), head-up verticalization 90° creates a large hydrostatic gradient (about 40 cm H_2_O) between the cranial (LV, CSS) and spinal (LSS) CSF space, preventing existence of a hypothetical unilateral CSF circulation from the ventricles to the cisterna magna (CM) and from there to LSS (Figure 2). In the case of aqueductal obstruction, that pressure gradient between the cranial and spinal space is maintained (Figure 2B). Following cervical stenosis, which interrupts hydrostatic CSF column, gradient becomes decreased (Figure 3) as it drops from 40 cm H_2_O (+36 cm H_2_O in LSS plus −4 cm H_2_O in LV) to 24 cm H_2_O (+28 cm H_2_O in LSS minus +4 cm H_2_O in LV). Thus, in neither of those cases do pressure gradients support the presumed unidirectional CSF circulation from the ventricles to the CM and to LSS.

Compliance is a ratio between the changes in CSF volume and CSF pressure expressed in mL/cm H_2_O. Distribution of craniospinal compliance is a fundamental question. A large number of studies were performed on animals and in humans in order to determine the contribution of individual cranial and spinal compliance to the total craniospinal compliance (Lofgren et al., 1973; Marmarou et al., 1975; Magnaes, 1989; Wahlin et al., 2010; Gehlen et al., 2017; Burman et al., 2018; Caton et al., 2021; Klarica et al., 2022).

It would be possible to determine the contribution of each individual compartment only if we separated the cranial from the spinal space (as ideally as possible) and then tested volume load-induced pressure changes inside each compartment. Thus, Marmarou has determined the cranial space contribution to be around 2/3 of the total compliance in his experiments on cats in which he separated the cranial from the spinal part of the CSF system at the cervical and the thoracic junction (Marmarou et al., 1975). Contrary to that, Lofgren et al. (1973) separated the cranial from the spinal space at the C_1_ level in dogs, and determined that the spinal compartment contribution is around 70%. Their results were more in line with the research from Magnaes who applied separate infusion boluses into the cranial and the spinal part of the CSF system in patients with cervical blockage (Magnaes, 1989). In our previous study, compliance was calculated in animals and in phantom after the addition/removal of a fluid volume from the spinal part of the system (Klarica et al., 2022). The calculation has been done for both horizontal and vertical positions. There was an exponential relationship between pressure changes and compliance. According to a well-known phenomenon, the value of compliance decreases with increasing CSF pressure, so this can be seen in our research. A similar phenomenon was also observed in patients during gravity-induced CSF pressure changes (Gehlen et al., 2017).

In this study, we have not performed volume changes by addition or substracion of fluid post surgically placed impairments inside the CSF system, so we cannot determine which compliance changes have been induced. However, based on the observed CSF pressure changes inside each individual CSF compartment, according to the well-known phenomenon, we can point to the direction of compliance changes. Therefore, if the pressure has increased, the compliance will decrease, and if the pressure has decreased, compliance will increase (Klarica et al., 2022).

### 4.3 Changes of cranial and spinal CSF pressure during head-down verticalization

In the head-down position at 270°, measurements of CSF pressures inside the LV, CSS and LSS in control animals with no CSF system impairment showed positive and similar pressure values inside the LV and CSS (around 40 cm H_2_O), which also corresponded to the hight of the total fluid column, i.e., to the distance between the lumbar space and cisterna magna (35–37 cm) with addition of the distance between cisterna magna and the cranial measuring cannulas (around 4 cm). In the case of Sylvian aqueduct obstruction, CSF pressures inside the LV, CSS and LSS remained similar to those obtained in animals without impairment (all of the measured values were somewhat higher than dose before aqueduct obstruction in the same position), which shows once again that there is no net CSF formation inside the isolated ventricles and no pressure gradient formation. It appears that in the head-down position there is a simultaneous transmission of pressure from the CSS in all directions across the larger surface of brain tissue to the smaller surface of isolated LV in the middle part of the brain, explaining gradient nonexistence. Similar effect was observed during mock CSF infusions into the CM at various rates in animals with aqueductal occlusion (Klarica et al., 2009). Infusions induced an increase of CSF pressure inside the subarachnoid space, which was instantly trasmitted across the brain tissue into the isolated ventricles, not resulting in gradient formation (Klarica et al., 2009). In fact, pressure gradient in those experiments developed only following infusions into isolated ventricles.

After insertion of cervical stenosis, CSF pressure inside the LV during head-down verticalization once again becomes positive. It appears that under higher pressure, a partial breach of stenosis occurs, causing hydrostatic pressure column to transfer partially from spinal subarachnoid space to cranial space (CSF pressure inside the LV in the head-down position at 270 ° was +38.9 cm H_2_O without stenosis and + 26.1 cm H_2_O with stenosis).

### 4.4 Clinical implications of our results

Since it is believed that CSF physiology in different animal species does not differ from that in humans, results of our research could provide an elucidation for many cases of various pathological conditions such as Arnold-Chiari malformations, spinal tumors, spinal haematomas, AV malformations or spinal oedema described in the literature related to concomitant hydrocephalus development in which the mechanism was described as unknown or unclear (Arseni and Maretsis, 1967; Raynor, 1969; Borgesen et al., 1977; Rifkinson-Mann et al., 1990; Cinalli et al., 1995; Morandi et al., 2006; Mirone et al., 2011; Wilson et al., 2013; Mukherjee et al., 2019; Mathkour et al., 2020). In the case of Arnold-Chiari type I malformation, the exact pathological mechanism for conjoined hydrocephalus development is still not clear. It is usually explained by primarily enlarged ventricles causing tonsilar prolaps through foramen magnum, or the prolaps causing an interruption of presumed unidirectional CSF circulation on the cranial basis level (Mukherjee et al., 2019; Mathkour et al., 2020). However, according to our results it seems that the process actually develops the other way around, i.e., that Arnold Chiari malformation primarily leads to impaired craniospinal CSF communication which in turn enables hydrocephalus development. This could also be corroborated by a number of literature data that shows variable results of surgical treatment (Mukherjee et al., 2019; Mathkour et al., 2020). Most often this implies foramen magnum decompression, however, not always succesful, and sometimes even causing the development of hydrocephalus that was not present prior to the procedure (Mukherjee et al., 2019). Thus, our results show major significance of the CSF system spinal compartment for the compensation of pressure and volume changes that occur inside the cranial space and how its stenosis for a number of possible reasons contributes to hydrocephalus development, particularly in an upright position in which we spend most of the time.

We also provide an explanation for the changes of CPP in different body positions during CSF pathway obstruction or stenosis on two different levels, with high and stable CPP in an upright position without any impairment that only slightly decreased in that same position after aqueductal blockade, but significantly decreases during cervical stenosis, which could, together with gradient development, also contribute to hydrocephalus development leading to tissue ischaemia. However, our results also show that aqueductal blockade by itself is insufficient to cause acute hydrocephalus development, especially in horizontal position, while in the vertical position only a slight gradient develops which is also unlikely to cause acute ventricular enlargement without any additional intracranial pathology. It is actually evident from this research that spinal pathological processes have much higher potential for acute hydrocephalus development than intracranial processes that block the aqueduct, primarily during the time we spend in an upright position. Thus, it would seem clinically important to perform a neuroradiological examination of the entire CNS system in the case of either spinal pathology or enlarged ventricles without other known intracranial causes.

## 5 Conclusion

In this research performed on cats as experimental animals, we have shown that CSF pressures measured inside the cranium gradually decrease below atmospheric level during animal head-up verticalization, and that there is no significant difference in the pressure values measured at the same hydrostatic level inside the LV and CSS. Additionally, we have found that experimentally caused aqueductal obstruction and subarachnoid space cervical stenosis do not induce CSF pressure gradient development during the horizontal plane measuring period. CSF pressure gradient develops only if the body position changes from horizontal to any other plane. These results suggest that cervical stenosis during the head-up vertical position can cause reduction of blood perfusion through the entire brain, and that aqueductal obstruction will only diminish the perfusion of the brain tissue surrounding the ventricles. Thus, observed phenomena imply that unimpaired cerebrospinal communication and preserved spinal space compliance could be evolutionary crucial for the development of bipedal walking as they enable optimal cerebral blood perfusion in any body position and prevent the development of pathophysiological changes which could in turn lead to hydrocephalus development.

## 6 Limitations

In this research, we have measured the changes of CSF pressure and observed the potential hydrocephalus development after experimentally induced impairment of CSF movement only during 15 min time periods following each change of the body position. We have not perfomed control phase measurements in the form of sham experiments since this would mean exposing cranial contets to atmospheric pressure. In order to avoid atmospheric pressure influence, we would have to hermetically close occipital craniectomy, than perform the control measurements, followed by reopening of occipital bone or cervical vertebrae in order to install stenosis or opstruction, than close the surgical field again. All of this would additionally damage the tissue (possible haemorrhage etc.) which could lead to experimental failure. Thus, we would have to additionally increase the number of experimental animals which goes against the RRR rules. Since during the experiments animals were not connected to ECG (it is techically challenging to record ECG during the changes of board and animal body positions), we were not able to measure heart frequency or amplitude of CSF pressure during cardiac cycle. However, the results are comparable to our previously published results obtained after similar experimentally caused CSF system stenosis or obstruction measured for longer periods of time, but mainly in one (mostly horizontal) body position. Since hydrocephalus development is usually a long-lasting (chronic) process, future investigations should strive to follow CSF pressure gradient changes in various pathological processes impairing normal CSF movement over much longer time periods.

## Data availability statement

The raw data supporting the conclusions of this article will be made available by the authors, without undue reservation.

## Ethics statement

The animal studies were approved by Ethics Committee of the University of Zagreb Medical School (Approval No. 04-76/2009-761). The studies were conducted in accordance with the local legislation and institutional requirements. Written informed consent was not obtained from the owners for the participation of their animals in this study because The owners were verbally informed about the experimental protocol which was previously approved by official Ethical committee (written consent form was not needed in that time).

## Author contributions

IJ: Data curation, Formal analysis, Investigation, Methodology, Writing – original draft, Writing – review & editing. DO: Conceptualization, Formal analysis, Funding acquisition, Project administration, Writing – original draft, Writing – review & editing. MR: Data curation, Formal analysis, Investigation, Writing – original draft, Writing – review & editing. KB: Formal analysis, Investigation, Methodology, Writing – original draft, Writing – review & editing. MK: Conceptualization, Data curation, Formal analysis, Funding acquisition, Investigation, Methodology, Project administration, Resources, Supervision, Validation, Writing – original draft, Writing – review & editing.
